# Editorial: Metainflammation and metabolism in hidradenitis suppurativa

**DOI:** 10.3389/fimmu.2026.1967931

**Published:** 2026-09-03

**Authors:** Nessr Abu Rached, Robert Sabat, John W. Frew, Falk G. Bechara

**Affiliations:** 1 Department of Dermatology, Bielefeld University, Medical School and University Medical Center East Westphalia-Lippe (OWL), Klinikum Bielefeld Rosenhöhe, Bielefeld, Germany; 2 International Centre for Hidradenitis Suppurativa/Acne Inversa (ICH), Department of Dermatology, Venereology and Allergology, Ruhr-University Bochum, Bochum, Germany; 3 Translational Skin Inflammation Research and former Psoriasis Research and Treatment Center, Department of Dermatology, Venereology and Allergology, Charité-Universitätsmedizin Berlin, Corporate Member of Freie Universität Berlin and Humboldt-Universität zu Berlin, Berlin, Germany; 4 Laboratory of Translational Cutaneous Medicine, Ingham Institute for Applied Medical Research, Liverpool, NSW, Australia; 5 Department of Dermatology, Liverpool Hospital, Liverpool, NSW, Australia; 6 School of Clinical Medicine, University of New South Wales Sydney, Sydney, NSW, Australia

**Keywords:** hidradenitis suppurativa, immunometabolism, metabolic syndrome, metainflammation, obesity

Hidradenitis suppurativa (HS) is a chronic, immune-mediated disease in which the inflammatory burden extends well beyond clinically visible lesions, such as inflamed nodules, abscesses, and draining fistulas in the skin (1). Its strong associations with obesity, insulin resistance, metabolic syndrome, cardiovascular disease, and metabolic dysfunction-associated steatotic liver disease support an understanding of HS as a systemic disorder rather than an isolated dermatosis (1). Furthermore, both cutaneous and extracutaneous alterations appear to contribute to the significant impairment of patients’ quality of life observed in HS (2). Metabolic and cardiovascular comorbidities in patients with HS also contribute significantly to the reduced life expectancy of those affected (1). Metainflammation—the persistent, low-grade immune activation generated by metabolic stress—may contribute to the systemic nature of HS. Metabolic dysfunction may amplify cytokine signaling, reshape immune-cell function and the tissue microenvironment, and contribute to fibrosis and treatment heterogeneity; conversely, chronic cutaneous inflammation may aggravate metabolic risk. This bidirectional relationship formed the rationale for the Research Topic “Metainflammation and Metabolism in Hidradenitis Suppurativa”.

The contributions to this Research Topic approach the immunometabolic continuum from complementary mechanistic, translational, and clinical perspectives. Moltrasio et al. synthesize evidence linking dysfunctional adipose tissue, altered adipokine profiles, insulin resistance, inflammasome activation, and TNF, IL-1, IL-6, and IL-17 signaling in HS. This review shifts metabolic comorbidity from the margins of HS care toward the center of disease biology and highlights therapeutic strategies that may interrupt self-reinforcing immune-metabolic loops. Chng et al. broaden this inter-organ perspective by examining shared immunological pathways connecting HS and psoriasis with metabolic dysfunction-associated steatotic liver disease. Together, these reviews argue for systematic metabolic and hepatic risk assessment while underscoring that causal pathways and treatment effects remain to be established prospectively.

The Research Topic also extends metabolic phenotyping beyond body mass index. In a computed tomography-based study, Balci et al. identify myosteatosis (the abnormal accumulation of fat within and around skeletal muscle), but not sarcopenia (the progressive loss of muscle strength, muscle mass, and physical function), as an independently associated metabolic feature of HS. This finding directs attention to muscle quality and ectopic fat distribution as potentially informative components of the HS metabolic phenotype. It also complements emerging evidence that dietary patterns, physical activity, and nutritional status may influence HS through metabolic, inflammatory, and microbiome-related pathways, although the available evidence remains heterogeneous and predominantly observational (3). Future studies should therefore integrate conventional cardiometabolic measures with body-composition assessment and longitudinal lifestyle data rather than treating adiposity as a single-dimensional exposure.

Two contributions illuminate the interface between systemic immune activity and the lesional microenvironment. Sia et al. discuss the TL1A-DR3 axis as a potential link among inflammation, fibrosis, tunnel formation, and extra-cutaneous comorbidity, and outline the therapeutic rationale for targeting this pathway. Casals-Diaz et al. report a reorganization of the CD4+ memory T-cell compartment in HS, with enrichment of central memory cells and marked tissue-specific proinflammatory responsiveness in lesions. Although the study cohort was small, the findings support a model in which circulating immune-cell dynamics and local tissue programming are connected but not equivalent. Thus, these manuscripts are also significant for the development of biomarkers. In fact, cellular markers in the blood appear to reflect recruitment processes that have already taken place (4). Meanwhile, lesional signals may better capture functional activation and therapeutic resistance.

Clinical heterogeneity is further emphasized by three contributions that challenge overly uniform models of HS. Lurel et al. describe patients of Moroccan origin with overlapping systemic autoinflammatory features and genetic or HLA findings, illustrating how ancestry, host genetics, and associated inflammatory phenotypes may inform individualized care. Yasuda et al. report refractory HS complicated by IgA vasculitis with nephritis that was controlled with surgery and bimekizumab, highlighting a possible convergence of neutrophilic and IL-17-mediated pathways while appropriately remaining hypothesis-generating as a single case (11). Finally, Delage et al. propose that, in at least a subset of patients, HS may be conceptualized as a host–microbiome or “autoinfectious” disorder arising from localized defects in immune control. This provocative perspective should stimulate testable studies of microbial persistence, barrier failure, host susceptibility, and treatment response. Interestingly, due to the significantly elevated local G-CSF concentrations in HS lesions, neutrophils there do indeed appear to respond more strongly to both bacterial products and metabolites (5).

Taken together, these articles reinforce three priorities. First, as previously recommended (1), metabolic, hepatic, and cardiovascular assessment should be integrated into HS care, with referral pathways tailored to individual risk. Second, disease stratification should combine clinical severity with immunological, metabolic, microbial, genetic, and body-composition features. Third, therapeutic studies should evaluate whether modifying metabolic dysfunction can improve both systemic health and cutaneous disease independently of weight loss. GLP-1 receptor agonists are a particularly promising example: early multicenter observational data suggest improvements in weight, quality of life, and some measures of HS activity, but the evidence remains non-randomized and does not yet distinguish weight-mediated from direct anti-inflammatory effects (6). Prospective controlled trials with standardized HS outcomes are therefore essential (7).

This Research Topic presents HS as a disease situated at the intersection of cutaneous inflammation, systemic immunometabolism, tissue remodeling, and host–microbe interactions. Its contributions do not support a single unifying key mechanism. Rather, they reveal biologically meaningful heterogeneity and a need for interdisciplinary, phenotype-guided research and, consequently, improved multidisciplinary care. This is urgently needed, as recent study shows that both patients and physicians are dissatisfied with the current medical care for HS (8). We hope that this Research Topic encourages studies capable of translating immunometabolic insight into earlier risk detection, more precise patient stratification, and therapies that address both the skin and the systemic burden of HS.
